# Evaluation of safety and affection of variable duration of dual antiplatelet therapy using aspirin plus ticagrelor after successful percutaneous coronary intervention for diabetic patients with acute coronary syndrome

**DOI:** 10.1186/s12872-026-05708-w

**Published:** 2026-03-27

**Authors:** Nour Eldeen Mahmoud Shabaan Ibrahim, Mohamed Tarek Mounir Zaki, Khaled Ahmed Fouad Abdel Magid, Khaled Ahmed Fouad, Mina Magued Abdalla Iskandar

**Affiliations:** Cardiology Department, Faculty of Medicine, Ain Shams University, Cairo, Ain Shams 11591 Egypt

**Keywords:** Ticagrelor monotherapy, Hemoglobin, Acute coronary syndrome

## Abstract

**Background:**

Atherosclerotic cardiovascular disease (ASCVD) represents the primary cause of morbidity and mortality among cases with diabetes.

**Purpose:**

To determine whether a strategy of ticagrelor monotherapy initiated after 3 months of dual antiplatelet treatment (DAPT) modifies the rate of major adverse cardiovascular effect (MACE) as the primary endpoint and bleeding events as the secondary endpoint, relative to a 12-month regimen of ticagrelor-based DAPT in diabetic cases with acute coronary syndrome (ACS) treated by Percutaneous coronary intervention (PCI).

**Methods:**

This randomized, open-label study included 400 diabetic patients with acute coronary syndrome (STEMI or NSTEMI) treated with PCI. Patients were randomly assigned to receive ticagrelor monotherapy after 3 months of DAPT or to continue ticagrelor plus aspirin for 12 months. The primary endpoint was major adverse cardiovascular events (MACE), and the secondary endpoint was bleeding events.

**Results:**

Hemoglobin level after three month was substantially higher in group 1, the mean ± SD was 12.97 ± 1.7 g/dl while group 2 was 12.6 ± 1.47 g/dl (*p* = 0.02). Platelet count did not differ markedly between groups. Similarly, the incidence of complications—mortality, chest pain, bleeding, ICH, ischemic stroke, recurrent MI, and repeated PCI—showed no significant variation between them. There was no significant difference in the incidence of MACE between the two groups during 12 months of follow-up. Ticagrelor monotherapy was associated with a lower incidence of overall bleeding events, mainly driven by reductions in mild bleeding, while rates of moderate or severe bleeding were comparable between groups.

**Conclusions:**

Switching to ticagrelor monotherapy after 3 months of DAPT results in a significant reduction in bleeding events among diabetic cases with ACS undergoing PCI, while maintaining a comparable safety profile regarding complications.

**Trial registration:**

Pan African Clinical Trials Registry (PACTR202511832264697) Date of registration 24 November 2025.

**Supplementary Information:**

The online version contains supplementary material available at 10.1186/s12872-026-05708-w.

## Introduction

Diabetes mellitus cases have a 2–3 times increased cardiovascular risk than those who do not have diabetes. Among people with diabetes, atherosclerotic cardiovascular disease (ASCVD) remains the dominant cause of morbidity and mortality, while DM can significantly heighten the likelihood of bleeding [1].

Primary PCI represents the preferred reperfusion strategy for cases with ST segment elevation myocardial infarction (STEMI) when performed within 12 h of symptom onset and achievable within 120 min of STEMI diagnosis by an experienced team. For non-ST-segment elevation myocardial infarction (NSTEMI), an immediate invasive approach within two hours of hospital arrival, with the intent of revascularization, is advised regardless of ECG or biomarker results [2].

According to current evidence, dual antiplatelet treatment (DAPT) reduces the incidence of stent thrombosis across the board, from acute to late occurrences.

Because continuous antiplatelet medication is linked to an increased risk of bleeding, this risk must be weighed against the potential benefit. The risk of bleeding in DAPT cases is proportional to the duration of the treatment [3].

The objective was to evaluate whether transitioning from DAPT to ticagrelor monotherapy at 3 months affects the rate of major adverse cardiovascular effect (MACE) as the primary endpoint, as well as bleeding events as the secondary endpoint, compared with maintaining ticagrelor-based DAPT for 12 months in diabetic cases with ACS who underwent PCI.

## Patients and methods

### Design and population

This was an interventional randomized study, performed on 400 diabetic cases who presented with ACS and were managed by PCI at the Faculty of Medicine, Ain Shams University, from November 2022 to November 2023. This randomized clinical trial was designed, conducted, and reported in accordance with the Consolidated Standards of Reporting Trials (CONSORT) 2010 guidelines.

The study protocol, including predefined endpoints and statistical analysis plan, was approved by the Scientific and Ethical Committee of the Faculty of Medicine, Ain Shams University (MD 307/2022) before initiation of patient enrollment. Trial registration in the Pan African Clinical Trials Registry (PACTR202511832264697) was completed retrospectively to ensure transparency and public availability of the trial data.

### Eligibility criteria

#### Inclusion criteria

Diabetic cases who received drug eluting stent implantation to treat ACS.

#### Exclusion criteria

Cases were excluded if they were < 18 or > 80 years, or if any condition increasing bleeding risk was present, including prior hemorrhagic stroke; ischemic stroke, dementia, or central nervous system impairment within one year; traumatic brain injury or brain surgery within 6 months; known intracranial tumor; suspected or confirmed aortic dissection; documented blood diseases; current oral anticoagulation therapy (AF patients); or mental retardation or mental illness. These exclusion criteria were applied to minimize bleeding risk and ensure patient safety during antiplatelet therapy.

The cases were randomized divided into two groups using computer randomization: Group I: Two hundred cases received ticagrelor monotherapy after 3-month DAPT till complete 12-month, group II (control group): Two hundred cases received ticagrelor plus aspirin DAPT for 12 months.

### Randomization and blinding

Eligible patients were randomly assigned in a 1:1 ratio using a computer-generated randomization sequence. Allocation was performed by an independent study coordinator not involved in patient enrollment or follow-up. Due to the nature of the intervention, the study was conducted in an open-label manner, and blinding of patients and treating physicians was not feasible.

### Treatment protocol

All patients received standard dual antiplatelet therapy consisting of aspirin (81–100 mg once daily) and ticagrelor (90 mg twice daily) for the initial 3 months following PCI. Thereafter, patients in Group I discontinued aspirin and continued ticagrelor monotherapy at a dose of 90 mg twice daily until 12 months, whereas patients in Group II continued dual antiplatelet therapy for the full 12-month period.

All patients underwent PCI using drug-eluting stents. The vascular access site was selected at the operator’s discretion and included transradial or transfemoral approaches. Lesion complexity was assessed according to the presence of multivessel coronary artery disease.

Medication adherence was assessed at each follow-up visit through structured patient interviews and pill count when available. Premature discontinuation and any treatment crossover were prospectively documented.

### Assessments

All cases underwent a complete clinical evaluation, including detailed history with particular focus on presenting symptoms and cardiovascular risk factors such as smoking history, HTN, DM, and other comorbidities. Diagnostic assessment included echocardiography, ECG, and laboratory investigations: cardiac markers (CK total, CK-MB, and cardiac troponins), complete blood picture with special attention to hemoglobin and platelet count, coagulation profile (INR, PT, PTT), complete lipid profile, renal function tests (urea and creatinine), liver enzymes (ALT and AST), and glycated hemoglobin (HbA1c).

### Outcome definitions

The primary outcome was major adverse cardiovascular events (MACE), defined as a composite of cardiac death, recurrent myocardial infarction, ischemic stroke, unstable angina requiring hospitalization, or repeat percutaneous coronary intervention.

Bleeding events were retrospectively adjudicated according to the Bleeding Academic Research Consortium (BARC) criteria to improve comparability with contemporary DAPT trials. Events originally recorded as trivial were classified as BARC type 1, mild bleeding as BARC type 2, moderate bleeding as BARC type 3a, severe bleeding as BARC type 3b, and life-threatening bleeding as BARC type 3c or type 5.

### Follow up

Follow-up assessments were performed at 3, 6, 9, and 12 months through scheduled outpatient clinic visits. Telephone follow-up was used when in-person visits were not feasible to assess symptoms, bleeding events, and major adverse cardiovascular events.

Every 3 months all cases were clinically examined to assess symptoms and any signs of hemorrhage and any MACE as unstable angina, recurrent myocardial infarction (MI), congestive heart failure (CHF), cerebrovascular accident, repeated PCI or cardiac death. Laboratory follow up CBC was performed every 3 months. All patients remained in contact with the research team throughout follow-up to deal with any medical events such as bleeding or MACE, and any events would be treated according to guidelines. Bleeding events was reported according to the severity of bleeding [4]. Trivial bleeding was defined as any bleeding episode that did not require medical evaluation or intervention, such as skin bruising or ecchymosis and self-resolving epistaxis.

Mild bleeding was defined as bleeding that required medical attention without hospitalization, including non–self-resolving epistaxis, genitourinary or upper/lower GIT bleeding without significant blood loss, or mild hemoptysis.

Moderate bleeding was defined as bleeding with significant blood loss (> 3 g/dl HB) and/or requiring hospitalization while the patient remained hemodynamically stable, such as genitourinary, respiratory, or upper/lower GIT bleeding with significant blood loss or requiring transfusion.

Severe bleeding was considered any bleeding event necessitating hospitalization and accompanied by severe blood loss (> 5 g/dl HB) in a hemodynamically stable patient, including severe genitourinary, respiratory, or upper/lower GIT bleeding.

Life-threatening bleeding was defined as severe active bleeding that presented an immediate threat to life, such as massive overt genitourinary, respiratory, or upper/lower GIT bleeding, or active intracranial, spinal, or ocular hemorrhage.

### Statistical analysis

Data were collected and tabulated, and all results were subjected to appropriate statistical analysis. Continuous variables were expressed as mean ± SD, and findings were subsequently presented in tables and discussed. Statistical analyses were exploratory in nature. Continuous variables were compared using Student’s t-test, and categorical variables using the chi-square test. The primary analysis followed the intention-to-treat principle. A secondary per-protocol sensitivity analysis excluding patients with premature treatment discontinuation was also performed. Missing data were minimal and were handled using complete-case analysis. Given the exploratory nature of repeated time-point and multiple endpoint testing, no formal multiplicity correction was applied. Accordingly, p-values for secondary and repeated analyses should be interpreted as hypothesis-generating. No formal a priori sample size or power calculation was performed. The sample size was determined pragmatically based on patient availability during the predefined study period. To account for baseline imbalances between the study groups, a multivariable logistic regression analysis was performed for bleeding outcomes at 12 months. The model included age, sex, chronic kidney disease, prior PCI, and hypercholesterolemia as covariates. In addition, given the observed baseline imbalances in hypercholesterolemia, prior PCI, chronic kidney disease, and target vessel distribution, clinically relevant covariates were included in the multivariable model to minimize residual confounding and improve causal interpretation.

## Results

**Fig. 1:**
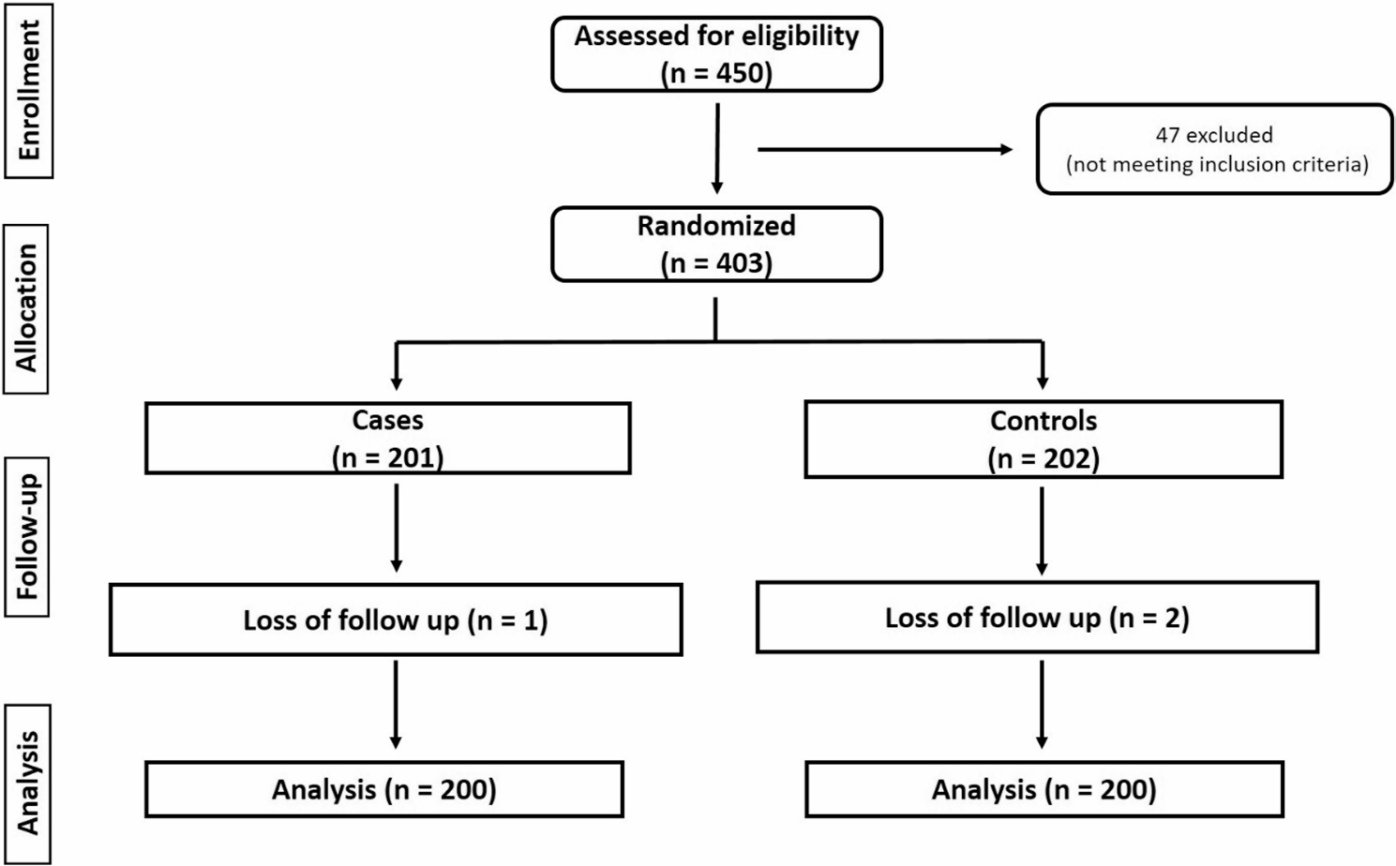
CONSORT flow diagram of patients

A CONSORT flow diagram illustrating patient screening, randomization, allocation, follow-up, and analysis is provided in Fig. 1. A total of 403 patients were randomized (201 and 203 per group). 3 cases lost follow up and the remaining 400 cases were included in the intention-to-treat analysis. Medication adherence exceeded 92% in both groups throughout follow-up. No treatment crossover between study strategies occurred.

Males constituted most of the studied population, accounting for 150 cases (75%) in group 1 and 138 cases (69%) in group 2. The average age of cases in group 1 was 59.88 ± 8.41 years, compared with 57.7 ± 7.8 years in group 2. Table 1.

**Table 1:** Distribution of demographic data between the studied groups

Parameter	Group 1 (*N* = 200)	Group 2 (*N* = 200)
Age (years)	59.88 ± 8.41	57.70 ± 7.80
BMI (kg/m²)	27.20 ± 3.78	26.98 ± 2.80
Sex		
Male	150 (75%)	138 (69%)
Female	50 (25%)	62 (31%)

Data are presented as mean ± SD, n (%), *BMI *body mass index

The percentage of cases with hypercholesterolemia was significantly higher in group1: 113 (56.5%), while in group 2: 85 (42.5%), with a p value ≤ 0.001. Also, cases with prior PCI were markedly higher in group 1: 48 (24%) while in group 2: 31 (15.5%), with a p value of 0.03. Regarding CKD, group 2 had a markedly higher number of cases 32 (16%), while group 1, it was just 18 (9%), with a p value of 0.03. There was no substantial variation between both groups regarding HTN, smoking status, prior MI, or atrial fibrillation. Table 2.

**Table 2:** History and comorbidities between the studied groups

Variable	Group 1 (*N* = 200)	Group 2 (*N* = 200)	χ²	*P* value
Current smoking	74 (37%)	69 (34.5%)	0.27	0.601
HTN	88 (44%)	79 (39.5%)	0.83	0.361
Hypercholesterolemia	113 (56.5%)	85 (42.5%)	7.84	≤ 0.001*
Prior MI	30 (15%)	36 (18%)	0.65	0.419
Prior PCI	48 (24%)	31 (15.5%)	4.56	0.030*
Chronic kidney disease	18 (9%)	32 (16%)	4.48	0.030*

*: P value < 0.05 is considered statistically significant, *χ²* Chi-square test, *DM *Diabetes mellitus, *HTN *Hypertension, *PCI *Percutaneous coronary intervention, *ACS *Acute coronary syndrome

As a target vessel, RCA was markedly higher in group 2 relative to group 1, while LCX + LAD and LCX + RCA as target vessels were substantially greater in group 1 with *p* ≤ 0.001. Also, PCI to RCA + LAD was notably higher in group 1 *P* = 0.003 Table 3.

**Table 3:** Comparison between numbers of lesions treated and target vessels between the studied groups

Variable	Group 1 (*N* = 200)	Group 2 (*N* = 200)	χ²	*P* value
Number of lesions treated				
1	119 (59.5%)	112 (56%)	0.50	0.470
2	40 (20%)	52 (26%)	2.03	0.150
> 2	41 (20.5%)	36 (18%)	0.40	0.526
Target vessel				
Right coronary artery (RCA)	41 (20.5%)	76 (38%)	14.79	≤ 0.001*
Left circumflex artery (LCX)	62 (31%)	75 (37.5%)	1.87	0.170
Left anterior descending artery (LAD)	49 (24.5%)	44 (22%)	0.35	0.550
RCA + LAD	13 (6.5%)	2 (1%)	8.38	0.003*
LCX + LAD	15 (7.5%)	0 (0%)	15.58	≤ 0.001*
LCX + RCA	18 (9%)	0 (0%)	18.84	≤ 0.001*
Left main + LAD	2 (1%)	0 (0%)	2.01	0.156
Left main	0 (0%)	3 (1.5%)	3.02	0.080
Multivessel CAD	48 (24%)	37 (18.5%)	1.81	0.180

*: P value < 0.05 is considered statistically significant,* χ²* Chi-square test, *CAD *coronary artery disease, *LAD *left anterior descending artery, *LCX *left circumflex artery, *RCA *right coronary artery

There was no statistically significant difference in the PCI approach between studied groups Table 4.

**Table 4:** PCI approach between the studied groups

Variable	Group 1 (*N* = 200)	Group 2 (*N* = 200)	χ²	*P* value
Trans-femoral approach	137 (68.5%)	152 (76%)	2.81	0.090
Trans-radial approach	63 (31.5%)	48 (24%)	2.01	0.156

*: P value < 0.05 is considered statistically significant, *χ²* Chi-square test, *PCI *Percutaneous coronary intervention

At the three-month follow-up, group 1 demonstrated a markedly higher hemoglobin level (12.97 ± 1.7 g/dl) compared with group 2 (12.6 ± 1.47 g/dl; *p* = 0.02). Platelet count showed no marked variation between groups. Likewise, no substantial variations were observed in the rate of complications, including mortality, chest pain, bleeding, ICH, ischemic stroke, recurrent MI, or repeated PCI Table 5.

**Table 5:** Outcome after 3 months between the studied groups

Variable	Group 1 (*N* = 200)	Group 2 (*N* = 200)	Test	*P* value
Platelet count (10³/ml)	238.7 ± 56.9	229.3 ± 38.9	t = 1.93	0.054
Hemoglobin (g/dl)	12.97 ± 1.70	12.60 ± 1.47	t = 2.33	0.020*
Mortality	0 (0%)	0 (0%)	χ² = 0	1.000
Chest pain	5 (2.5%)	6 (3%)	χ² = 0.09	0.760
Bleeding				
Trivial	30 (15%)	27 (13.5%)	χ² = 0.18	0.660
Mild	14 (7%)	19 (9.5%)	χ² = 0.82	0.360
Moderate	3 (1.5%)	2 (1%)	χ² = 0.20	0.650
Severe	0 (0%)	1 (0.5%)	χ² = 0.32	0.320
Total bleeding	47 (23.5%)	49 (24.5%)	χ² = 0.20	0.650
ICH	0 (0%)	0 (0%)	χ² = 0	1.000
Ischemic stroke	0 (0%)	0 (0%)	χ² = 0	1.000
Recurrent MI	0 (0%)	0 (0%)	χ² = 0	1.000
Repeated PCI	1 (0.5%)	0 (0%)	χ² = 1.00	0.320

*: P value < 0.05 is considered statistically significant, *D* Standard deviation, *t* t-test, *χ²* Chi-square test, *MI* Myocardial infarction, *ICH* Intracerebral hemorrhage, *PCI* Percutaneous coronary intervention

After 6 months, group 2 demonstrated a notably lower platelet count (221.4 ± 29) than group 1 (237.9 ± 51.3; *P* ≤ 0.0001). Hemoglobin level did not differ markedly between groups. Furthermore, the incidence of complications—mortality, chest pain, bleeding, ICH, ischemic stroke, recurrent MI, and repeated PCI—was comparable between both groups. Table 6.

**Table 6:** Outcome after 6 months between the studied groups

Variable	Group 1 (*N* = 200)	Group 2 (*N* = 200)	Test	*P* value
Platelet count (10³/ml)	237.9 ± 51.3	221.4 ± 29.2	t = 3.95	≤ 0.0001*
Hemoglobin (g/dl)	13.01 ± 1.48	12.80 ± 1.40	t = 1.46	0.150
Mortality	0 (0%)	1 (0.5%)	χ² = 1.00	0.320
Chest pain	5 (2.5%)	3 (1.5%)	χ² = 0.51	0.480
Bleeding				
Trivial	22 (11%)	23 (11.5%)	χ² = 0.03	0.870
Mild	17 (8.5%)	18 (9%)	χ² = 0.03	0.860
Moderate	2 (1%)	4 (2%)	χ² = 0.68	0.410
Severe	0 (0%)	1 (0.5%)	χ² = 1.00	0.310
Total bleeding	41 (20.5%)	51 (25%)	χ² = 1.41	0.230
ICH	0 (0%)	0 (0%)	χ² = 0	1.000
Ischemic stroke	1 (0.5%)	0 (0%)	χ² = 1.00	0.310
Recurrent MI	2 (1%)	3 (1.5%)	χ² = 0.20	0.650
Repeated PCI	2 (1%)	2 (1%)	χ² = 0	1.000

*: P value < 0.05 is considered statistically significant, *SD *Standard deviation, *t* t-test, *χ²* Chi-square test, *MI *Myocardial infarction, *PCI *Percutaneous coronary intervention, *ICH *Intracerebral hemorrhage

Group 2 demonstrated a markedly lower platelet count (mean ± SD: 219.5 ± 22.46) relative to group 1 (232.2 ± 46.3; *p* ≤ 0.001). Mild bleeding was substantially higher in group 2, with 41 cases (20.5%) affected, versus 23 cases (11.5%) in group 1 (*p* = 0.01). Recurrent chest pain occurred in five cases (2.5%) in group 1, while no cases were observed in group 2 (*p* = 0.02). No statistically marked variation was found between the studied groups regarding hemoglobin level or complications such as mortality, trivial and moderate bleeding, ICH, ischemic stroke, recurrent MI, or repeated PCI. Table 7.

**Table 7:** Outcome after 9 months between the studied groups

Variable	Group 1 (*N* = 200)	Group 2 (*N* = 200)	Test	*P* value
Platelet count (10³/ml)	232.2 ± 46.3	219.5 ± 22.5	t = 3.42	≤ 0.001*
Hemoglobin (g/dl)	13.04 ± 1.47	12.80 ± 1.14	t = 1.82	0.070
Mortality	1 (0.5%)	0 (0%)	χ² = 1.00	0.320
Recurrent chest pain	5 (2.5%)	0 (0%)	χ² = 5.06	0.020*
Bleeding				
Trivial	18 (9%)	29 (14.5%)	χ² = 2.92	0.080
Mild	5 (2.5%)	11 (5.5%)	χ² = 2.34	0.120
Moderate	0 (0%)	1 (0.5%)	χ² = 1.00	0.320
Severe	0 (0%)	0 (0%)	χ² = 0	1.000
Total bleeding	23 (11.5%)	41 (20.5%)	χ² = 6.02	0.010*
ICH	0 (0%)	1 (0.5%)	χ² = 1.00	0.320
Ischemic stroke	0 (0%)	1 (0.5%)	χ² = 1.00	0.310
Recurrent MI	1 (0.5%)	2 (1%)	χ² = 0.34	0.560
Repeated PCI	1 (0.5%)	1 (0.5%)	χ² = 0.34	0.560

*: P value < 0.05 is considered statistically significant, *SD *Standard deviation, *t *t-test, *χ²* Chi-square test, *MI *Myocardial infarction, *PCI *Percutaneous coronary intervention, *ICH *Intracerebral hemorrhage

At 12 months, ticagrelor monotherapy was associated with a clinically meaningful absolute reduction in any bleeding of 9.0% compared with continued DAPT (8.0% vs. 17.0%; risk difference − 9.0%, 95% CI − 15.3 to − 2.7; *p* = 0.006). The reduction was primarily driven by mild bleeding (absolute risk reduction 4.0%). Rates of moderate or severe bleeding were low and comparable between groups.

No deaths, ischemic strokes, recurrent myocardial infarctions, or repeat revascularizations were observed in either group during follow-up (0.0%, 95% CI 0.0–1.9% for each outcome). Chest pain occurred in 1.0% (95% CI 0.3–3.6%) of the monotherapy group versus 0.0% (95% CI 0.0–1.9%) of the DAPT group (*p* = 0.156) Table 8.

**Table 8:** Outcome after 12 months between the studied groups

Variable	Group 1 (*N* = 200)	Group 2 (*N* = 200)	Test	*P* value
Platelet count (10³/ml)	230.4 ± 45.5	231.1 ± 27.7	t = 0.19	0.852
Hemoglobin (g/dl)	13.01 ± 1.50	13.10 ± 1.14	t = 0.68	0.499
Mortality	0 (0%)	0 (0%)	χ² = 0	1.000
Chest pain	2 (1%)	0 (0%)	χ² = 2.01	0.156
Bleeding				
Trivial	14 (7%)	23 (10.5%)	χ² = 2.41	0.120
Mild	2 (1%)	10 (5%)	χ² = 5.49	0.010*
Moderate	0 (0%)	1 (0.5%)	χ² = 1.00	0.320
Severe	0 (0%)	0 (0%)	χ² = 0	1.000
Total bleeding	16 (8%)	34 (17%)	χ² = 7.40	0.006*
Ischemic stroke	0 (0%)	0 (0%)	χ² = 0	1.000
Recurrent MI	0 (0%)	0 (0%)	χ² = 0	1.000
Repeated PCI	0 (0%)	0 (0%)	χ² = 0	1.000

*: P value < 0.05 is considered statistically significant, *SD* Standard deviation, *t* t-test, *χ²* Chi-square test, *MI *Myocardial infarction, *PCI *Percutaneous coronary intervention

After adjustment for baseline imbalances, ticagrelor monotherapy after 3 months of DAPT was independently associated with a significantly lower risk of any bleeding at 12 months compared with continued dual antiplatelet therapy (adjusted OR 0.22, 95% CI 0.11–0.44; *p* < 0.001).

In a sensitivity analysis focusing on clinically relevant bleeding (mild or higher), the association remained significant after adjustment for chronic kidney disease, age, sex, and prior PCI (adjusted OR 0.073, 95% CI 0.016–0.344; *p* < 0.001) (Table 9).

**Table 9:** Multivariable logistic regression analysis for bleeding at 12 months

Variable	Adjusted OR	95% CI	*P* value
Ticagrelor monotherapy	**0.22**	0.11–0.44	< 0.001
Chronic kidney disease	5.21	2.35–11.55	< 0.001
Age (per year)	1.03	0.99–1.07	0.165
Male sex	1.08	0.55–2.13	0.816
Prior PCI	2.44	1.28–4.65	0.007
Hypercholesterolemia	2.03	1.07–3.87	0.031

## Discussion

Aspirin represents the cornerstone therapy for coronary artery disease. Nevertheless, recurrent ischemic events continued to occur in some cases treated with aspirin monotherapy, prompting the development and use of additional antithrombotic agents, particularly in high-risk cases [5].

In our study, analysis of demographic data revealed that males represented most of the study population, accounting for 150 cases (75%) in group 1 and 138 cases (69%) in group 2. The mean age of cases in group 1 was 59.88 ± 8.41 years, whereas in group 2 it was 57.7 ± 7.8 years.

Supporting our observations, Lee et al. [6] showed that most of the cases with ACS who underwent PCI were males (about 77%), with a mean age of 60 ± 4.6 years.

In our study, smoking, hypercholesterolemia, and hypertension were the most prevalent comorbidities.

In agreement with the current study Han et al. [7] revealed that HTN, hypercholesterolemia and smoking were the most common comorbidities among cases who underwent PCI.

In our study more than half of the cases (86% of the cases) had one vessel treated, and the most common target vessel was LCX followed by RCA then LAD and about one-fifth of cases (21% of the cases) had multivessel CAD.

In contrast to the current study, Osman et al. [8] showed that most common target vessel was LAD followed by RCA then LCX. As well, Han et al., [7] reported that the most common target vessel was the LAD, followed by the RCA and then the LCX, and that approximately two thirds of cases had multivessel CAD.

In our study, more than two-thirds of the cases (72%) underwent the transfemoral approach.

In contrast to Lee et al. [6] and Lee et al. [9] who treated most of their cases via transradial approach.

A systematic review and meta-analysis by Anjum et al. [10] showed that trans-radial approach had better outcome and less bleeding complications compared to transfemoral approach.

In our study, transfemoral approach was more used due to availability of femoral sheaths and operators’ preference.

Our study demonstrated that there was no marked variation between both groups regarding platelet count or complications, including mortality, chest pain, bleeding, ICH, ischemic stroke, recurrent MI, and repeated PCI.

However, hemoglobin level after three months was higher in monotherapy group, the mean ± SD was 12.97 ± 1.7 g/dl while DAPT group was 12.6 ± 1.47 g/dl (*p* = 0.02). It was noted that both groups had slightly reduced Hb level.

At the 6-month follow-up, the DAPT group showed a significantly reduced platelet count, with a mean ± SD of 221.4 ± 29, whereas the monotherapy group recorded a mean ± SD of 237.9 ± 51.3 (*P* ≤ 0.0001).

This finding may be explained by the use of aspirin in the DAPT group. van Diemen et al. [11] recently showed that aspirin intake induces platelet inhibition and decreases reticulated platelets, with evening administration producing greater inhibition and a more pronounced reduction compared with morning dosing. Therefore, aspirin use may contribute to lowering platelet count.

In our study, follow-up after 9 months showed that platelet count remained significantly lower in the DAPT group (mean ± SD: 219.5 ± 22.46) compared with the monotherapy group (mean ± SD: 232.2 ± 46.3; *p* ≤ 0.001).

The observed differences in platelet counts between the two groups may be explained by the pharmacological effects of aspirin. Aspirin irreversibly inhibits cyclooxygenase-1, leading to suppression of thromboxane A₂–mediated platelet activation and a reduction in reticulated (young) platelets. Prolonged aspirin exposure has been associated with lower circulating platelet counts, which may explain the modest but consistent reductions observed in the DAPT group at 6 and 9 months. Importantly, these laboratory differences were not accompanied by clinically significant bleeding or ischemic events.

Total bleeding was markedly higher in DAPT group as 41 cases had episodes of bleeding (20.5%, mainly trivial to mild, with only 1 case having moderate bleeding), while in monotherapy group, 23 cases had episodes of bleeding (11.5%), *p* = 0.01. so Ticagrelor monotherapy was associated with a reduction in overall bleeding events, mainly driven by differences in mild bleeding, while rates of moderate or severe bleeding were comparable between the two groups.

Recurrent chest pain was more frequently reported in the monotherapy group at 9 months (5 cases vs. no case). Although this finding favored continued DAPT, it was not accompanied by objective ischemic endpoints such as myocardial infarction, ischemic stroke, or repeat revascularization. Given the low event rates, this observation should be interpreted as hypothesis-generating and warrants further investigation in larger studies.

Nevertheless, the hemoglobin level was comparable between the studied groups, with no significant statistical variation.

No marked variation was detected between the groups in terms of complications such as mortality, ICH, ischemic stroke, recurrent MI, or repeated PCI. However, the absence of a statistically significant difference in ischemic endpoints should not be interpreted as evidence of non-inferiority. Given the low event rates observed and the overall sample size, the study was not powered to detect small differences in MACE, and a type II error cannot be excluded.

At the 12-month follow-up, the DAPT group exhibited notably higher rates of mild and total bleeding (*p* < 0.05), whereas the incidence of moderate and severe bleeding was comparable between both groups. Similarly, platelet count, hemoglobin level, and complication rates—including mortality, ICH, ischemic stroke, recurrent MI, and repeated PCI—showed no marked variations.

The observed absolute risk reduction of 9% in overall bleeding at 12 months corresponds to a number needed to treat (NNT) of approximately 11 patients to prevent one bleeding event. This magnitude of effect suggests potential clinical relevance, particularly in diabetic ACS patients who are intrinsically at higher bleeding risk.

These findings support the beneficial role of ticagrelor monotherapy following 3 months of DAPT in cases undergoing PCI. Compared with continued DAPT, ticagrelor monotherapy in diabetic cases post-PCI substantially reduced bleeding at 9 and 12 months and improved platelet count at 6 and 9 months, without elevating ischemic event rates.

Consistent with the present results, Han et al. [7] analyzed 1,169 cases randomized at Chinese sites in the TWILIGHT trial and demonstrated that ticagrelor monotherapy led to a significant reduction in clinically relevant bleeding, with no increase in ischemic complications, compared with ticagrelor plus aspirin in high-risk PCI cases.

Similarly, Lee et al. [9] conducted a randomized trial involving 2,980 high bleeding risk cases with ACS who underwent PCI and found that ticagrelor monotherapy after 3-month DAPT resulted in fewer adverse clinical events—including major bleeding and adverse cardiac and cerebrovascular events—compared with 12-month ticagrelor-based DAPT, regardless of bleeding risk criteria.

Similarly, Wang et al. [12] conducted a meta-analysis evaluating long-term ticagrelor monotherapy in cases with T2DM following PCI. They reported that long-term ticagrelor monotherapy following a short duration of DAPT was correlated with a notably lower risk of MACE and all-cause mortality. There were no significant differences in cardiac death, MI, stroke, stent thrombosis, onducted a randomized trial involving 2,980 high bleeding risk cases with ACS who underwent PCI and found that ticagrelor monotherapy after 3-month DAPT resulted in fewer adverse clinical events—including major bleeding and adverse cardiac and cerebrovasculaor repeated revascularization. Ticagrelor monotherapy was also linked to a significantly lower risk of TIMI-defined minor or major bleeding compared with DAPT.

Large randomized trials such as GLOBAL LEADERS and STOPDAPT-2 have explored strategies of early aspirin discontinuation following PCI. While these trials differed in patient selection, P2Y12 inhibitors used, and duration of dual antiplatelet therapy, their overall findings support the concept that selected patients may safely transition to P2Y12 inhibitor monotherapy with reduced bleeding risk and no excess ischemic events. Our findings in diabetic ACS patients are directionally consistent with this growing body of evidence.

In contemporary practice, antithrombotic therapy after PCI is increasingly individualized according to procedural complexity, ischemic burden, and bleeding risk. Recent evidence emphasizes the importance of tailoring DAPT duration, particularly in complex PCI settings where the balance between thrombotic and hemorrhagic risk is delicate [13]. Within this evolving framework, our findings support the concept that selected diabetic ACS patients may benefit from early aspirin discontinuation while maintaining ischemic protection.

These findings are aligned with contemporary guideline recommendations. The 2023 European Society of Cardiology (ESC) guidelines for the management of acute coronary syndromes support antiplatelet de-escalation strategies, including early aspirin discontinuation and P2Y12 inhibitor monotherapy, in selected patients at increased bleeding risk.

Atrial fibrillation was infrequent in our cohort and patients requiring chronic oral anticoagulation were excluded. In patients with AF undergoing PCI, antithrombotic management is substantially more complex, often requiring combination therapy with oral anticoagulants and antiplatelet agents to balance ischemic and bleeding risks. Contemporary evidence emphasizes individualized strategies in this setting [14]. Therefore, our findings should not be extrapolated to patients with AF who require long-term anticoagulation.

Moreover, our findings are consistent with the systematic review and meta-analysis by O’Donoghue et al. [15], which evaluated the safety and efficacy of aspirin discontinuation in 32,145 cases receiving a P2Y12 inhibitor after PCI. The authors demonstrated that stopping aspirin 1–3 months post-PCI reduced major bleeding risk by 40% compared with continued dual antiplatelet therapy, without increasing the incidence of MACE, MI, or death.

In diabetic cases, P2Y12 inhibitor monotherapy was correlated with a lower risk of MACCE and resulted in a favorable reduction in major or minor bleeding compared with standard DAPT. In non-diabetic cases, P2Y12 inhibitor monotherapy significantly reduced major or minor bleeding events without increasing the risk of MACE. These findings suggest that P2Y12 inhibitor monotherapy can reduce bleeding events without elevating the risk of stent thrombosis or MI in the general population.

The magnitude of bleeding reduction was substantially greater in the non-DM population than in those with DM. Notably, P2Y12 inhibitor monotherapy demonstrated the unexpected benefit of lowering MACCE risk among DM cases.

In line with our findings, Sadeghi et al. [16] performed a systematic review and meta-analysis assessing ticagrelor monotherapy following short-term DAPT in diabetic and non-diabetic cases after PCI. Their results demonstrated a lower incidence of major bleeding in both diabetics and non-diabetics, with the greatest benefit observed among non-diabetic cases. For cardiovascular outcomes, ticagrelor monotherapy significantly reduced cardiac death in the diabetic group, whereas this reduction was not statistically significant in non-diabetics compared with the standard 12-month DAPT. Moreover, rates of MI and ischemic stroke did not significantly differ with short-term DAPT.

Although baseline differences were observed in hypercholesterolemia, prior PCI, chronic kidney disease, and target vessel distribution, adjusted analyses confirmed that ticagrelor monotherapy remained independently associated with lower bleeding risk. Nevertheless, residual confounding cannot be entirely excluded.

The limitations of this study include the small sample size, being a single center study and relatively short follow up period. Further multicenter randomized controlled trials with larger population and longer follow up periods are recommended.

## Conclusions

Ticagrelor monotherapy after 3 months of DAPT significantly reduced bleeding events, without increasing the risk of intracerebral hemorrhage, ischemic events, MI or mortality in ACS cases. These findings support that P2Y12 inhibitor monotherapy, after 3 months of DAPT, is an alternative choice of medical treatment, for diabetic cases with ACS and undergoing PCI.

### Limitation

This single-center, open-label study has several limitations, including retrospective trial registration, baseline imbalances in hypercholesterolemia, prior PCI, chronic kidney disease, and target vessel distribution despite randomization, and lack of power for ischemic endpoints. The exploratory statistical approach, non-standard bleeding classification, and absence of time-to-event analyses limit causal inference. Low event rates may further reflect selection of relatively lower-risk patients. Also, one possible limitation is that the lack of a statistically significant difference in the study may be due to the low event rate and the high risk of a type II error, as the sample size was determined based on the number of patients available during the study period.

The absence of time-to-event analyses represents a methodological limitation. Due to very low event rates and absence of recurrent ischemic events, survival modeling was not feasible. Future larger trials with higher event rates should incorporate Kaplan–Meier and Cox regression analyses to better characterize temporal risk patterns.

## Supplementary Information

Supplementary Material 1.

## Data Availability

The data that support the findings of this study are available on request from the corresponding author.

## Abbreviations

ACS: Acute coronary syndrome
PCI: Percutaneous coronary intervention
DAPT: Dual antiplatelet therapy
MACE: Major adverse cardiovascular events
DM: Diabetes mellitus
CKD: Chronic kidney disease
HTN: Hypertension
ICH: Intracerebral hemorrhage
MI: Myocardial infarction
