# Supplementary material for: Evaluation of safety and affection of variable duration of dual antiplatelet therapy using aspirin plus ticagrelor after successful percutaneous coronary intervention for diabetic patients with acute coronary syndrome
Source: BMC Cardiovasc Disord. 2026 Mar 27;26:300. doi: 10.1186/s12872-026-05708-w (PMC13064407; doi:10.1186/s12872-026-05708-w)
Supplement: Supplementary file 1 — Supplementary Material 1. [file 12872_2026_5708_MOESM1_ESM.docx]

# CONSORT 2010 checklist for randomized trials

Trial: Evaluation of Safety and Affection of Variable Duration of Dual Antiplatelet Therapy Using Aspirin plus Ticagrelor after Successful Percutaneous Coronary Intervention for Diabetic Patients with Acute Coronary Syndrome

**Registration: Pan African Clinical Trials Registry (PACTR202511832264697)**

| **Section/Topic** | **Item No.** | **CONSORT checklist item** | **Reported on page/section** |
| --- | --- | --- | --- |
| **Title and abstract** | 1a | Identification as a randomized trial in the title | Title |
|  | 1b | Structured summary of design, methods, results | Abstract |
| **Introduction** | 2a | Scientific background and rationale | Introduction |
|  | 2b | Specific objectives or hypotheses | Introduction |
| **Methods – Trial design** | 3a | Description of trial design | Design and population |
|  | 3b | Changes after commencement | Not reported |
| **Methods – Participants** | 4a | Eligibility criteria | Eligibility criteria |
|  | 4b | Settings and locations | Design and population |
| **Methods – Interventions** | 5 | Interventions for each group | Design and population |
| **Methods – Outcomes** | 6a | Primary/secondary outcomes defined | Follow up |
|  | 6b | Changes to outcomes | Not reported |
| **Methods – Sample size** | 7a | Sample size determination | Not reported |
|  | 7b | Interim analyses | Not applicable |
| **Randomization – Sequence** | 8a | Method used | Computer randomization |
|  | 8b | Type of randomization | Not specified |
| **Allocation concealment** | 9 | Mechanism used | Not reported |
| **Randomization – Implementation** | 10 | Who generated and assigned | Not reported |
| **Blinding** | 11a | Who was blinded | Not reported |
|  | 11b | Similarity of interventions | Not applicable |
| **Statistical methods** | 12a | Statistical comparisons | Statistical analysis |
|  | 12b | Additional analyses | Not reported |
| **Results – Participant flow** | 13a | Numbers analyzed | Results tables |
|  | 13b | Losses/exclusions | Not reported |
| **Recruitment** | 14a | Recruitment/follow-up dates | Design and population |
|  | 14b | Reason trial ended | Not reported |
| **Baseline data** | 15 | Baseline table | Tables 1–2 |
| **Numbers analyzed** | 16 | Denominators | Tables 5–8 |
| **Outcomes** | 17a | Outcome results | Tables 5–8 |
|  | 17b | Absolute and relative effect sizes | Absolute only |
| **Ancillary analyses** | 18 | Additional analyses | None |
| **Harms** | 19 | Harms/unintended effects | Tables 5–8 |
| **Discussion** | 20 | Limitations | Discussion |
|  | 21 | Generalisability | Discussion |
|  | 22 | Interpretation | Discussion |
| **Other information** | 23 | Registration number | Abstract, Clinical trial number |
|  | 24 | Access to protocol | Not reported |
|  | 25 | Funding | Funding section |
